# A Case of Hepatotoxicity After Receiving a COVID-19 Vaccine

**DOI:** 10.7759/cureus.20455

**Published:** 2021-12-16

**Authors:** Muath M Alqarni, Ammar Z Faloudah, Amjad S Alsulaihebi, Hassan K Halawani, Abdulmajeed S Khan

**Affiliations:** 1 Department of Medicine, Umm Alqura University, Makkah, SAU; 2 Department of Internal Medicine, Hera General Hospital, Makkah, SAU

**Keywords:** drug-induced acute liver failure, covid19 vaccine, vaccine side effects, coronavirus disease, hepatotoxicity

## Abstract

The coronavirus disease 2019 (COVID-19) has led to a global health crisis. Its clinical manifestations are well-documented, and severe complications among patients who survived the infection are being continuously reported. Several vaccines with well-established efficacies and excellent safety profiles have also been approved. To date, few side effects of vaccines have been reported. Drug-induced hepatotoxicity is an extremely rare side effect of these vaccines, with few reported instances. In this case report, we describe a patient who experienced hepatotoxicity after receiving the COVID-19 vaccine from Pfizer BioNTech.

## Introduction

The coronavirus disease 2019 (COVID-19) has caused an unprecedented global health crisis. Its most common symptoms include fever, cough, fatigue, and myalgia. Rarely, patients may develop an acute respiratory distress syndrome or multiple organ failure [1]. Other conditions, such as liver injury, may occur. Various factors can lead to liver injury, including severe inflammatory responses, severe hypoxia, drug-induced liver injury (DILI), and worsening of pre-existing metabolic conditions [2]. The manifestations of liver injury vary from elevated serum levels of aspartate aminotransferase (AST), alanine aminotransferase (ALT), and bilirubin to hepatic dysfunction in severe cases [3]. In May 2020, the Pfizer‐BioNTech COVID‐19 vaccine received emergency authorization for use among adolescents aged 12-15 years [4]. Clinical trials have demonstrated that its efficacy in this age group may be as high as 100%. The vaccine’s side effects are typically mild and non-life-threatening, including headache, fatigue, myalgias, and chills [5]. However, there have been reports on extremely rare yet life-threatening side effects, such as anaphylactic shock, deep venous thromboembolism, and pulmonary embolism [1,6].

## Case presentation

A 14-year-old female, not known to have any chronic illnesses, presented to the emergency department with epigastric pain, diarrhea, nausea, and vomiting for the past four days. Three days prior to her current presentation, the patient received the second dose of the Pfizer/BioNTech BNT162b2 mRNA COVID-19 vaccine. The patient denied the use of any pharmaceutical, herbal, or recreational drugs. Upon arrival to the emergency room, the patient had a temperature of 36.9°C, a pulse rate of 128 bpm, a blood pressure of 90/63 mmHg, a respiratory rate of 18 rpm, and oxygen saturation of 97% on room air. On physical examination, the patient was conscious, oriented, and had a Glasgow coma scale (GCS) score of 15/15. In addition, she had mild epigastric tenderness and jaundice. No signs of chronic liver disease were evident.

**Figure 1:**
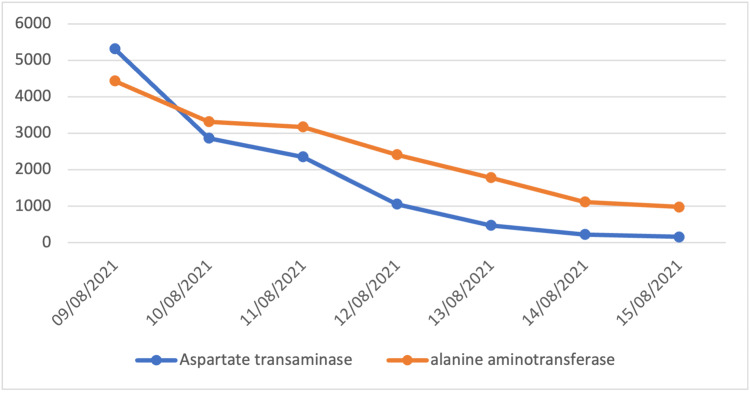
AST and ALT trends Normal ranges: AST - Aspartate transaminase (0-40 U/L), ALT - Alanine transaminase (0-41 U/L)

**Figure 2:**
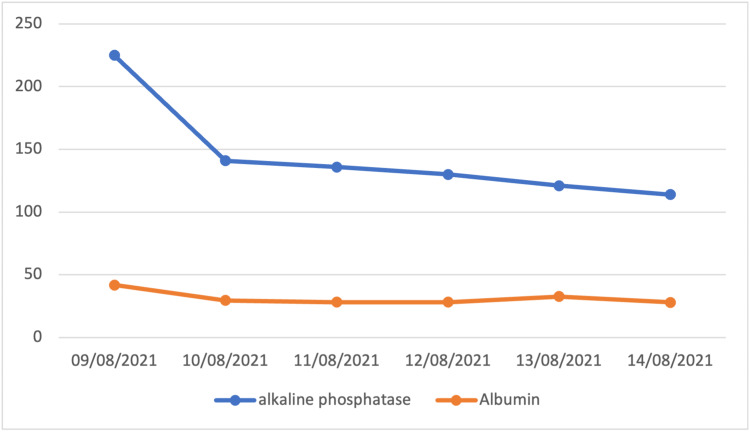
Alkaline phosphatase and albumin trends Normal ranges: Albumin: 39.7-49.4 mmol/L, Alkaline phosphate: 35-104 mmol/L

On the first day of admission, vital signs returned to normal after resuscitation with intravenous fluids. The patient's urine was dark as observed after urinary catheter insertion. The hematology panel showed Leukopenia, neutropenia, and lymphopenia among others as seen in Table 1. Biochemical and coagulation profile workups are shown in Table 2. Abdominal ultrasound was unremarkable except for a minimal rim of free fluid in the pelvic cavity. Along with conservative treatment, the patient was started on N-acetylcysteine, lactulose, and Vitamin K. In addition, ceftriaxone was given as an empirical antibiotic. On the second day, the results of AST, ALT, and alkaline phosphatase decreased, yet remained abnormally high (Figures 1, 2).

**Table 1:** Trend of the complete blood counts and bilirubin Normal ranges: White blood cells: 4-10 x 10^9^/L, Neutrophils: 2-7x10^3^/µL (40%-75%), Lymphocytes: 1-3.5x10^3^/µL (20%-45%), Platelets: 150-400x10^3^/µL, Total bilirubin: 0-21 µmol/L, Direct bilirubin: 0-3.4 µmol/L

Date	White blood cells	Neutrophils	Lymphocytes	Platelets	Total bilirubin	Direct bilirubin
09/08/2021	1.67	0.9 (53.9%)	0.68 (40.7%)	107	121.1	86.1
10/08/2021	1.22	0.58 (47.6%)	0.56 (45.9%)	107	117.9	81.1
11/08/2021	1.08	0.37 (34.3%)	0.66 (61.1%)	101	156.6	94.3
12/08/2021	1.25	0.49(39.2%)	0.69 (55.2%)	101	179.6	106.8
13/08/2021	1.09	0.53(48.6%)	0.53 (48.6%)	86	213.4	122.2
14/08/2021	1.00	0.52 (52.0%)	0.45 (45.0%)	87	231.6	154.0
15/08/2021	1.38	0.72 (52.2%)	0.60 (43.5%)	83	291.4	187.5

**Table 2:** Trends of the chemical and coagulation profiles Abbreviations: APTT: Activated Partial prothrombin time, INR: international normalized ratio Normal ranges: Prothrombin time: 11-13 seconds, Partial prothrombin time 28-40 seconds, INR: 0.9-1.2, Potassium: 3.5-5.1 mmol/L, Sodium: 136-145 mmol/L, Ammonia: 11-51 µmol/L

Date	Prothrombin time	APTT	international normalized ratio	Potassium	Sodium	Ammonia	Creatine
09/08/2021	57.9	53.2	4.61	5.53	134	162.1	36
10/08/2021	48.817	49.948	5.46	3.97	125	211.8	9
11/08/2021	34.832	53.109	3.691	3.86	132	156.4	20
12/08/2021	25.030	50.602	2.516	3.41	139	38.8	26
13/08/2021	23.932	64.209	2.388	4.65	137	80.4	51
14/08/2021	23.6	47.5	1.81	3.44	133	125.7	16
15/08/2021	15.371	42.298	1.429	3.33	131	32.5	11

On the fourth day, the patient became agitated and non-responsive, when assessed, her GCS score dropped to 8/15. Consequently, she was transferred to the intensive care unit, where she was intubated. Consultations from gastroenterology, infectious disease, neurology, and hematology departments were requested. Following this, a wide range of infectious, immunological, and toxicological tests were ordered (Tables 3,4). Nevertheless, all the results were unremarkable. To rule out structural brain pathologies, a brain computed tomography without contrast was performed. A suspicious hypodense lesion in the right temporal lobe was identified. However, the findings from the brain magnetic resonance imaging were unremarkable.

**Table 3:** Immunologic and infectious work-up for liver disease Abbreviations: Ig: immunoglobulin, PCR: polymerase chain reaction

Test	Result
Blood culture and sensitivity	Negative
Cytomegalovirus immune globulin M (CMV IgM)	Negative
Indirect Coombs test	Negative
Direct Coombs test	Negative
Hepatitis A virus immune globulin M (HAV IgM)	Negative
Hepatitis C virus antibodies (enzyme immunoassays)	Negative
Hepatitis B surface antigen (HBsAg)	Negative
Urine culture and sensitivity	Negative
human immunodeficiency virus serology (HIV)	Negative
Stool Culture and sensitivity	Negative
Chikungunya PCR	Negative
Alkhurma virus PCR	Negative
Dengue virus PCR	Negative
Dengue virus serotype	Negative
Dengue virus IgG	Negative
Dengue virus nonstructural protein 1 (NS1)	Negative
Dengue virus IgM	Negative
Rift valley fever PCR	Negative
Anti-Smooth Muscle Antibody (ASMA)	Negative
Antinuclear Antibodies (ANA)	Negative
Anti-Liver-Kidney Microsomal Antibody (LKM)	Negative

**Table 4:** Urine and blood toxicology panel

Name of the tested substance	Result
Paracetamol	Negative
Salicylic acid	Negative
Narcotic alkaloids and its derivatives	Negative
Benzodiazepines	Negative
Barbituric acid	Negative
Tricyclic antidepressants	Negative
Organophosphorus pesticides	Negative
Ethanol	Negative

The patient's level of consciousness returned to normal by the seventh day, her liver enzyme levels continued to decline, and her symptoms have resolved. Afterward, she was transferred to a liver transplant center for further investigation and management.

## Discussion

DILI is the most common cause of acute liver injury in developed countries [7]. Its presentation ranges from an incidental elevation of liver enzymes to outright acute liver failure [8]. There are two types of DILI: idiosyncratic and intrinsic. The most common type of which is the intrinsic type that has a short latency period and is dose-dependent. An example of an offending agent in this type is acetaminophen. Contrarily, the idiosyncratic type is less common and has a longer latency. A few examples of idiosyncratic drugs are amoxicillin, nonsteroidal anti-inflammatory drugs, and isoniazid [9]. In our case, we hypothesized the type of DILI to be idiosyncratic, due to the short latency period.

The diagnosis of DILI is made by identifying a relationship between drug exposure and the onset of liver disease. It is important to exclude any infectious, autoimmune, or other forms of liver disease. A thorough medical history and a high clinical suspicion are the basis for a correct diagnosis. A recovery following withdrawal from an offending agent may indicate DILI [10]. A diagnostic criterion that can be utilized in diagnosing DILI is the Rousse Uclaf Causality Assessment Method of the Council of International Organization of Medical Science (RUCAM/CIOMS) [11]. This criterion was applied to our patient's case, and a total of 6 was calculated, indicating that DILI is probable.

Currently, there is no effective treatment for DILI other than discontinuing the offending drug and providing patients with supportive measures until their condition improves [12]. The exception is acetaminophen intoxication in which an antidote can be used in management, namely N-acetylcysteine. Early transfer of patients with idiosyncratic DILI to tertiary liver centers is important. Liver transplantation increases overall survival from 27.8% to 66.2% [13]. Withholding the transplantation can result in infection, brain damage, organ failure, and even death [14].

There have been three reports of patients having hepatic failure, with one case being acute, after receiving the Pfizer/BioNTech BNT162b2 mRNA vaccine in the United Kingdom between September 12, 2020 and September 4, 2021. Moreover, there have been 17 reported cases of liver injury, with two cases being drug-induced [15]. The possible side effects of the COVID-19 vaccines on the liver are not limited to one type. Two case reports suggested that the ChAdOx1 nCoV-19 vaccine (Oxford-AstraZeneca) may trigger acute autoimmune hepatitis [16]. Mann et al. reported a case of a 61-year-old female who developed generalized weakness and low-grade fever after receiving the second dose of Pfizer/BioNTech BNT162b2 mRNA vaccine. The patient had an ALP of 207 µ/L, total bilirubin of 6.2 mg/dL, direct bilirubin of 3.9 mg/dL, a WBC of 17 x 10^9^, and AST of 37 U/L. All laboratory workup and imaging to investigate possible etiologies were unremarkable. As compared to our case, there were significant differences in age group, initial presentation, and degree of liver injury [17].

Prior to her recent presentation, our patient had no chronic illnesses. Given that her history, physical examination, and laboratory workups were unremarkable, the patient's clinical picture was attributed to hepatotoxicity secondary to the Pfizer/BioNTech BNT162b2 mRNA vaccine, the only pharmacological agent that she was exposed to before her current presentation.

## Conclusions

This is a case of hepatotoxicity in a 14-year-old patient that occurred after receiving the second dose of the Pfizer/BioNTech BNT162b2 mRNA vaccine. The exhaustive clinical and laboratory evaluation failed to establish any other plausible etiology besides the vaccine. The purpose of this report is to raise awareness of this uncommon but potentially life-threatening side effect.
